# Life Quality in Care Homes: Chinese and Danish Older Adults’ Perspectives

**DOI:** 10.1007/s12144-022-02921-6

**Published:** 2022-02-27

**Authors:** Puxiang Ren

**Affiliations:** Department for the Study of Culture, University of Southern Denmark, Campusvej 55, 5230 Odense, DK Denmark

**Keywords:** Older adults’ wellbeing, Happiness/wellbeing, Life quality, Care homes, Interpretative phenomenological analysis, Cross-cultural comparative study

## Abstract

The demand for care homes appears to be emerging as a key future trend in response to the burgeoning population of older adults, with the need for care provision increasing accordingly. Life quality, happiness and well-being are important factors associated with the care of older residents. This qualitative study explores how older adults moving into care homes view their life quality, from their own perspectives, in two quite different cultural contexts, Chinese and Danish. Older care residents in Shanghai and Denmark participated in the study by means of semi-structured and in-depth interviews. An interpretive phenomenological analysis approach was used for data analysis. Four interrelated themes were identified: positive transfer; positive environment; positive capability and positive experience. The findings demonstrated that older adults considered their quality of life as the result of a dynamic process. Their pursuit of a harmonious status, centered on “change” as the core value, which encompassed both the simplicities and complexities of life. Both older adult groups cared more about their emotional wellbeing, which focused mainly on positive emotions being stimulated while negative emotions were shunned. In the situations when they were “harmonized” by society systems, there was an important emotional thread which continued throughout their whole life that was strongly associated with life quality which was the relationship with family members – be it in the past, present or future.

## Introduction

The burgeoning population of ageing older adults is well recognized. In China, by 2050, the number of adults over 65 years old is projected to account for 26.9% of the total population, according to a recently announced report (National Statistical Bureau of the People’s Republic of China, 2019). Similarly, in Denmark, it is estimated that this cohort will reach up to 25% of the Danish population by 2040 (Mandag Morgen, 2010). These numbers crystallize the picture that societies are ageing, which at the same time highlights issues that are causing concern. Furthermore, the pace at which this change is occurring is particularly rapid in China, as compared to developed western countries (Chu & Chi, 2008).

Alongside this, the demand for long-term care is escalating (Eurostat, 2016). Institutional-based care has been the dominant form of long-term care for the elderly who need care the most in western nations, whereas this is a growing tendency in China (Feng et al., 2011). The rationale behind this can be an increased dependency on others due to a decline in cognition, the prevalence of acute health crises (Johnson & Bibbo, 2014), insufficient provision of specialized care in community-based settings (Kao et al., 2004), and a disproportionate decrease in the reliance on the traditional home-based informal care which engenders new forms of outside assistance to be explored. On the whole, these have been viewed to be as a result of profound demographic shifts and socioeconomic changes (Gu et al., 2017) – the shrinking proportion of the younger generation, as well as the burgeoning of small nuclear families and “empty-nest elders” (Chu & Chi, 2008; Feng et al., 2011).

In addition to these changes, which themselves are associated with a degree of uncertainty, the relocation to a nursing home may be viewed as a huge existential challenge for most older adults, as it implies the abandonment of the old and familiar, but stable, homeplace and life patterns. Research suggests that old people may fear entering care homes more than dying itself, as they presume that their inner needs will often not be met by the environment (De Bellis, 2010). Newer residents often tend to vocalize their feelings of being abandoned and isolated – be it by family or society (Yeboah et al., 2013; Yu et al., 2016). Others are likely to experience complex emotional adjustments, with the emergence of a suspicious understanding of the world, and self (Sullivan, 2017). What is more distressing is that these negative experiences are likely to give rise to psychological effects, such as loneliness and depression, which in some cases, score higher than for elders in community dwellings (Ron, 2004). Consequently, this is associated with a higher than normal level of suicidal thoughts (Kaneko et al., 2007), and additionally, with the increase in the prevalence of dementia comes the loss of many fundamental human qualities (Baltes & Smith, 2003). On the surface, this cohort in care homes appear to have a different quality of life than other older adults, and what constitutes a “good life” seems to be a counter-intuitive and counter-stereotypical account. It is necessary for us to fuel growing concerns on the quality of life of this cohort as with the increasing elderly population, comes an increase in demand for care homes.

It is suggested that culture should be taken into consideration when assessing the quality of life among older adults in nursing homes, as it may account for variations (Albertini et al., 2007; Bekhet & Zauszniewski, 2014). In China, for example, living with their adult children and receiving family all-round care are the most desired expectations that are traditionally valued by ageing parents. The familism and collectivism are a reflection of the Confucian values of “filial piety” (Chu et al., 2011). On the other hand, institutional care facilities have been historically reserved for “the Three No’s, those with no children, no income and no relatives” (Chen, 1996). Furthermore, the practice of filial piety is currently ensured by law, in that “children who have come of age have the duty to support and assist their parents” (Wu et al., 2005; Yang, 1988). As such, it seems that the transition to nursing home care is a contradiction to the entire social cultural expectations of family caregiving and a violation of filial piety obligations (Chang & Schneider, 2010), and thus might explain the strong negative association with the quality of life perceived there by the elderly.

As is the norm in countries with Confucian traditions, family solidarity and mutual dependency are more significant than individualism and privacy, and supportive families have long been identified as a condition for happiness, even though it can be provided without co-residence. Some elders have claimed in verbatim that the change in their living arrangements into care homes was not their decision, which has led to variations in their integration and wellbeing after they moved there (Wu et al., 2009). Other research supports the view that Chinese cultural patterns of values such as harmony, balance and collectivism have somewhat hampered the residents’ ability to establish new relationships with their peers and staff members; in addition, elders often downplayed the importance of issues such as autonomy, lack of privacy and the like (Lee, 1999; Lee et al., 2002), and hence they experienced a more tedious, monotonous and lonely life (Chuang & Abbey, 2009). These might be strongly associated with a negative impact on their quality of life.

In seemingly individualistic cultures, there are still expectations in terms of family support (though mostly of emotional support), with those who need care being most likely to receive it from care homes or through home care. As a result of modernization, the emergence of the modern welfare state has developed hand-in-hand with the nuclear family and has impeded intergenerational family interactions (Albertini et al., 2007). Despite that, certain forms of family support do still exist. In European cultures, there generally exists a net downward flow of both financial and social support from the older to the younger generations, rather than vice versa (Albertini et al., 2007), and maybe a consideration that affects older people’s wellbeing when transferred to care homes, coupled with the reduced contact from their family. Therefore, there is now also a movement in Denmark and other western countries to attempt to re-establish intergenerational contact (Hernandez et al., 2020; Tapper, 2019; Vang, 2020). The transfer to care homes, where routines and harmony are usually overweighed, and the values of independence, practical autonomy, rationality, and personal decisions which are cherished by individualism can often be strongly limited (Kehyayan et al., 2015). Consequently, this has led to some (Higgs & Gilleard, 2015) to suggest that residential care homes can be regarded as a negative lifestyle choice. Although there is a growing awareness of the culture change movement in nursing homes, which aims to boost the quality of life of residents by focusing on person-centered care, an empirical base has yet to be generated (Zimmerman et al., 2014). The proportion of older people in care home suffering from mental health issues such as depression, remains high. According to one report (Jongenelis et al., 2004), almost 8.1% of nursing home residents suffer from major depression, 14.1% suffer from minor depression while a further 24% has sub-clinical depression, despite advances in care facilities.

Although there have been countless studies conducted on the nursing home experiences of older people in western countries, they were usually carried out from particular perspectives, such as languages, religion, and the like (Krizaj et al., 2018). Thus, there remains a paucity of research available on the quality of life in elderly care homes. Besides, the studies that have been performed have almost exclusively been limited to a single country and focused on variables such as social relationships with fellow residents, autonomy, social support, and the like (Bergland & Kirkevold, 2005; Cooney et al., 2009; Custers et al., 2012). Little research has been devoted to the quality of life of the elderly in care homes in different cultural contexts. Therefore, the focus of this article is to present a comparative study which aims to address this gap by exploring how older people moving into care homes view their quality of life, from their own perspectives, and exploring it from two quite different cultural contexts, Chinese and Danish. The former implying a cultural expectation towards family connection and other characteristics associated with collectivism, whereas the latter indicating preferences towards individualism, in which values linked with autonomy and differentiation are emphasized. Some have argued that countries with different cultural backgrounds are not comparable due to their complexity attributes (Yan et al., 2014), however, it should be seen as an opportunity to investigate the similarities and differences instead of an obstacle to performing such a comparative study.

Earlier studies have indicated that the care home environment can be detrimental to the well-being of older adults (Tuckett, 2007), and focused mainly on the conceptions of quality of life or a good life based on psychological theory. However, these studies were often confined to predetermined variables which may have limited the ability to gain an understanding of what a quality life means from both a holistic and in-depth view. This study aims to bridge that gap by directly asking care residents questions such as, “What do you think is a good life?,” “How does your life compare to your idea of a good life?,” and “Tell me about a recent situation where you felt especially good or bad?” This should provide a better basis for gaining an understanding of what matters for the quality life for older adults.

## Perception of Life Quality

In this paper, “life quality” is used in a holistic sense, a comprehensive understanding of how older adults themselves understand life quality in the context of care homes—a “view from the inside.” This construct covers both “hedonic” wellbeing (typically known as subjective wellbeing in empirical studies), and a more objective understanding of the “good life,” which is in congruent with optimal functioning championed by positive psychologists (Keyes, 2005, 2007; Deci & Ryan, 2008; Seligman, 2011).

There is a large amount of research on wellbeing in general and a good life in social science literature, noticeably in psychology. Psychologists have attempted to associate the good life with two distinct yet overlapping approaches – hedonic wellbeing and eudemonic wellbeing (Deci & Ryan, 2008), in which the former defines the good life in terms of happiness, life satisfaction and “affective balance” – a hybrid notion of subjective wellbeing (SBW) (Diener et al., 2002). However, the criteria for a good life in SBW approach has remarkably been predetermined by researchers rather than by the elderly themselves, as researchers intended to measure it with self-reported life satisfaction (Pavot & Diener, 2008) as well as the “day reconstruction method” (Kahneman et al., 2004). By contrast, the eudemonic wellbeing approach refers to the good life as being more concerned with meaning, values and self-realization, which may or may not be accompanied by feeling good or having one’s preference satisfied (Ryan & Deci, 2001; Urry et al., 2004). Yet, there is the train of thought that these may serve to confuse, rather than clarify what comprises a good life in old age (Meeks et al., 2010), on the presumption that their definition was formed from a series of predetermined variables that were measured on the basis of current psychological theories, such as the emphasis on self-development or self- expression and these might still appear to reflect other constructs of a successful life (Delle Fave et al., 2011).

Hence, psychologists have depicted the good life as an optimal functioning in its zenith by uniting the above mentioned two approaches (Keyes, 2007; Park & Peterson, 2009; Seligman, 2011). However, the vast majority of existing research has generally targeted young and middle-aged cohorts, with relatively few studies focusing on older adults, let alone those living in care homes. There is the possibility that the elderly in care homes may hold a more sophisticated understanding in terms of what constitutes a good life than those identified in extant studies.

In contrast to more dominating approaches, this study focused on giving priority to the older adult’s own understandings and particular psychological dispositions, conceiving wellbeing holistically. Klausen (2019) painted a rich theoretical picture of older adults’ wellbeing, which emphasized older people’s tendency to give priority to proximal variables in their current environment, and their capability to obtain wellbeing via adjusting their judging standards as well as by controlling their emotions. The qualitative methodology described in the next section was chosen because it avoids imposing any specific notion of wellbeing and is sensitive to the differences and subtleties of the older adults’ own understanding (including differences and subtleties due to cultural factors, which are especially pertinent to a comparative study like this). It also serves to complement existing wellbeing research, which is predominantly based on life satisfaction surveys. In keeping with the methodological approach, the study was not hypothesis-driven, but the holistic and process-oriented (Klausen, 2018) understanding of wellbeing, as well as the critical view of standard notions of a good life in old age, has served as theoretical background and informed the interpretation of the findings.

## Methodology

Qualitative studies provide a detailed insight into the many complexities associated with factors that influence quality of life, from the perspective of older adults (Stathi & Simey, 2007). This study will focus on comparing the life quality of older adults in care homes in China and Denmark for the following reasons. Firstly, both countries have very similar ageing populations, not only in the proportion of their ageing populations, but also in the tendency and necessity of them needing to move into care homes, as well as similar degrees of urbanization, though the rural–urban divide may still be more significant in China than in Denmark, and institutionalized care has a longer history in Denmark. Secondly, from a cultural perspective, both countries appear to have strikingly conflicting values in terms of collectivism versus individualism, and different perspectives on family life. Hence, it seems prudent to discover whether older adults share any similarities when moving to seemingly comparable care homes, although as the national contexts differ, it may help to understand how culture-specific factors influence the quality of life perceived by them. Some researchers (Bengtson et al., 2003; Palmore, 1983) have stated that the proportion of the ageing population, the extent of urbanization, as well as culture are essential variables to consider when carrying out cross-cultural comparative research on ageing.

The core of this study is based on semi-structured interviews and open-ended questions, which compared older adults in care homes in terms of the life quality in China and Denmark. The data from Denmark was based on eight semi-structured interviews, with participants aged between 74–95 years, conducted in a total of five municipal elderly care units. The interview guide devised was based on preliminary impressions from field observations at the care home. However, due to limitations imposed by the Covid-19 pandemic, we were unable to carry out as comprehensive a study in China as had been originally planned. A total of nine interviews, with participants aged between 74–95 years, in a care home in Shanghai were conducted. All the participants involved in this study were selected by care home managers. Purposeful sampling (Patton, 2002) was adopted for the selection of older adults, after taking into account their age, gender, educational levels and whether they were cognitively capable of communicating. We are cognizant that to some extent there may be shortcomings with this asymmetrical methodological approach.

All the participants involved in the interviews were informed that participation was voluntary, and would not in any way negatively impact the quality of their care. Participants were asked questions regarding their values in terms of what contributes to better life quality, if they felt they experienced quality of life at present, and in what ways their caregivers were aware of the status of the participants’ life quality. Interview questions included “What do you think is a good life?,” “How does your life compare to your idea of a good life?,” “Tell me about a recent situation where you felt especially good or bad?,” and “How do you think the staff become aware of your day to day needs?” The interviews were conducted in Mandarin and Danish, as appropriate, and subsequently translated into English. In addition, a back-translation was made to check the validity. Each interview lasted between 30 to 60 min and were recorded with the informed consent of the participants, and then fully transcribed verbatim for analysis. The interviews were conducted in a quiet room and pseudonyms used throughout to protect the privacy of the participants.

Interpretative phenomenological analysis (IPA) was adopted to allow the real-life experiences that the residents associate with life quality to be explored, and shared practices and common meanings to be uncovered (Smith, 2009, 2015). Transcripts were read meticulously several times to obtain a complete holistic perspective, from which notes were made to ensure interpretations remained true to the participants’ accounts, as far as possible. This was accompanied by reflexion on the researchers’ attitudes and assumptions, as IPA not merely requires researchers to ‘bracket’ preconceptions, but also implies a “hermeneutic” understanding (Smith, 2009, 2015), recognizing preconceptions as ineliminable and not necessarily sources of bias, but something that has to be dealt with reflexively (for a deeper discussion on such methodological issues, see (Emiliussen et al., 2021)). Common emergent themes were identified based on these notes, which were further refined, condensed and categorized.

## Findings

The interviews provided by the participants revealed four interrelated key themes: positive transfer (positive attitude as a good start; being mate with the room); positive environment (geographical living environment; policy environment); positive capability (physical health; enjoyment of food; learning to compromise); positive experience (previous life experience matters; agency; respect and emotional health). Each of these themes were further divided into several subthemes that influenced one another.

### Positive Transfer

Taylor (1992) describes how an authentic act (where ‘authentic’ positive normative connotation) can be seen as an expression of an individual’s underlying attitudes and genuine responses to feelings. This may indicate that a positive attitude can predict a positive transfer for older adults when moving into care homes.

### Positive Attitude as a Good Start

Older residents were asked about their perceptions of the quality of life in care homes, and a prevailing theme that emerged was that a positive attitude (willingness to adapt) when transferring into a care home appeared to have a positive impact on their life quality after their relocation. This could be viewed as a change of abandoning traditional ways of life and embracing a new space. A positive attitude was considered by most participants as the most difficult to maintain, but also necessary and meaningful, as it might be conducive to the subsequent adaptation to their “new homes”, as well as to their positive psychological state. This was particularly apparent in the Chinese group. As recounted by one elder who emphasized the importance of adopting a proactive approach to life:

“If one willingly moves to a care home, he/she would be likely to easily accept the status quo and learn to adapt to the new environment including positively taking part in different activities. Otherwise, it would have been easy to think about it too much, which typically occurs in many older Chinese women. But I think, nowadays, changes are happening rapidly, it’s very important to be adaptable to the environment.” Female, 85 years. Chinese.

Whilst the Chinese participants may traditionally view staying in their family homes as the ideal scenario, there is an increasing willingness to positively accept relocating to care homes. This is not merely as a means of minimizing loneliness and/or emptiness in the family home, and avoiding potential conflicts with family members,^1^ but also as a way of avoiding being a burden to their adult children which may give rise to psychological distress with feelings of guilt and shame. The Chinese participants also viewed it as a means of maintaining family harmony. All these factors may stimulate positive emotions, albeit in different ways and to varying degrees. Although for some, this may be an attempt to avoid negative emotions, others are more pragmatic, as recounted by one female resident who described new social contacts in the care home as a positive experience:

“I love warm places with many people, and I used to be lonely at home. My children were busy, and I rarely saw them. So, I moved here and now I am busy meeting new people. I feel I have more energy now.” Female, 83 years. Chinese.

This highlights that it is not purely about avoiding the negative consequences of living alone that is important. It also tallies with the assumption by Haybron (2008) that emotions play a constitutive role in one’s wellbeing, in particular positive emotions outweighing negative ones. It can impart a sense of hope for the elderly, especially if they have experienced deteriorating health that has required assistance coupled with a perceived lack of support from family. They also viewed a positive attitude regarding the relocation as a personal responsibility for them to ensure they made the best of their life.

“I was willing to move to a care home, because of my fractured legs. I disliked being a burden to my children. They were busy with work and their kids’ education every day. And I believe you must have heard that ‘no filial son at the bedside, in cases of chronic sickness’. To avoid potential conflicts among us, I prefer to be in a care home. Now, I am happy in the care home, being looked after by staff here. My adult children are happy too.” Female, 83 years. Chinese.

“As to moving to care homes, some old people’s thinking and concepts are old-fashioned. But, I am open-minded and don’t care about others’ comments. I was actively open to moving, and I like the services here.” Female, 91 years. Chinese.

The Danish residents shared a similar view, that positive acceptance of a relocation, and support from family members might be beneficial to later life in a new home, although they did not share the same concerns as their Chinese peers due perhaps to their cultural differences.

“So, my daughter thought that now it was the time to move, as last winter I had a bad cold and things like that when I was alone up “there” (previous place of residence) then, so it seems now I should…” Female, 94 years. Dane.

### Being mate with room

In contrast, it appears that those with a more passive attitude in accepting the transition, an unwillingness to move, may have instilled a strong sense of self-imposed constraints, which may negatively affect their life quality. The consequences of adhering to stereotypical perceptions lead to feelings such as the fear of being forced to live in a public domain which is not recognized as being home and give rise to a sense of lack control of their current life, both physically and psychologically. This may impose a greater risk on the physical health of older adults, as reflected in findings by Ohrnberger et al. (2017), where the past mental health of individuals had a significant impact, direct and indirect, on one’s physical health.

“If [I had been] opposed to relocation, this would be another case. Like my cousin, who is in her eighties, pretty conservative and prefers to be taken care of by her family members, and was forced to relocate to a care home by her adult children. The result is pretty negative: she ate little, disapproved of the facility, and refused all the help after the relocation. Every day she merely sat in her room alone, staring out of the window and having nothing to do. Finally, this exacerbated her health.” Female, 85 years. Chinese.

This is consistent with the findings of Wu et al. (2009), where relocation to care homes was viewed as a process of “forced choice” where the older adults were victims of both the influence of traditional culture and modernization. The process not only invokes fear due to the loss of the previous familiar network, with personal emotions attached (as the older adults now have less access to their families and friends), “*I miss my children, especially grandchildren and feel sad about not seeing them grow up*,” as one female Chinese resident mentioned, but also feelings of frustration towards the communication barriers encountered in their new surroundings. The inability to build new lasting relationships in the new environment can consequently lead to bottled-up emotions and feelings of being trapped in a prison, until one might succumb to some alarming physical symptoms^2^ – like a “rage virus” in the body. Despite the older residents living with many other people in the care home, a feeling might remain of having no belonging or attachment to the home in one’s own mind. A male resident depicted it as follows:

“The nursing home is like a public place. Older people come and leave as they wish. There is no sense of home living here. To me, I am single and have no family members. All I want to do is merely survive here, nothing else. I don’t often talk with others, I mostly stay in my room watching TV.”Male, 85 years. Chinese.

This was similarly supported by another resident when she described her unwillingness to relocate to a care home as: “*I am scared to know that one suddenly dies nearby.” Female, 84 years. Chinese.*

They viewed themselves as being homeless^3^ and abandoned, and thus rarely participated in group activities or engaged in conversations with others. The consequences of not acknowledging care homes as home, albeit involuntarily, can thus be negative. It is likely to regularly impact on the residents’ daily choices and behaviors, and lead to increasingly stronger feelings of isolation and loneliness, which tended to affect one’s quality of life and wellbeing. This was found to equally apply to older Danes who frequently expressed psychological resistance when moving to care homes, either due to internal reasons, such as a fear of lack of autonomy, or an external reason such as feeling it was an unsatisfactory arrangement enforced on them by society to which, with no alternative options, they merely were expected to accept.

When questioned about whether he felt he had a good life in care homes, a 95-year-old male Danish care resident responded with: “*and I didn't have anywhere to go……But it's not the home here… the home here*” which is another example demonstrating how the older resident disliked living in a care home, in this case after being frequently refused by the hospitals. In some cases, it’s even worse:

“There’s this lady, who’s in her 70’s, who by her daughters’ will, now moved to (part of town) from down by the harbor. She hadn’t been outside for a while, and I think that she actually didn’t want to move, and that she’s angry over having been moved. She’s getting thinner, she isn’t getting anything and she doesn’t take her medicine. So her daughters are extremely worried. But she simply does not want us to come (and help). She simply does not want it.” Female, 74 years. Dane.

This is in line with an earlier study by Lee et al. (2002) which found that the older adults’ negative stereotypes of aged care were associated with their sense of well-being before their relocation, which intensified their subsequent suffering after moving into the care homes. Although unwilling to move to a communal environment, due to their increasing lack of abilities, some have no choice but to move to live with a group of strange people and a life that may be viewed as consisting of a number of regularly repeated daily trivial rituals. Unlike the Chinese cohort, where possible anxiety and concerns emerged as the biggest fear, the older Danes feared lack of privacy the most. The current collective life in the nursing homes draws a clear contrast with their previous life experience, where the emphasis is mostly on a more private life. However, they often counteracted these negative expressions with “*I cannot complain*” which was frequently uttered by many care residents. This phrase might be taken to implicitly suggest a certain level of dissatisfaction with life in care homes. Such “adaptive” reactions have been associated with self-abnegation or self-delusion by Sen (1999). However, they could also be viewed as genuine maintenance of life quality, a more positive kind of “hedonic adaptation” which enables having a good life with really nothing to complain about, adjusting expectations and standards to what the person actually has or can realistically achieve. This has been pointed out by Klausen (2019), who states that older adults’ positive self-assessment of wellbeing can partly be explained by adaptation, especially in difficult circumstances, and that it can sometimes be seen as genuinely positive. Brandtstädter (2015) likewise argues that as individuals age, strategies of assimilative and accommodative coping may be utilized to foster resilience.

Despite relocating to care homes, the older adults still desired to maintain connection with their family members so that they would not be alienated from those with whom they were most closely connected emotionally. Both the Chinese and the Danish group seem to pursue harmony, with the former focusing mostly on family-centered harmony and the latter on social systems harmony.^4^

### Positive Environment

Positive environment decribes the environment in which the older adults are situated. This includes not only the geographical living environment, but also the institutional environment which may affect, directly and indirectly, quality of life. In this theme, life quality could be reflected with both easy access to the family home and acceptance that the institutional environment provides them with a sense of security for smoothing any potential challenges in the nursing home. According to attachment theory, attachment patterns not only provide individuals with spiritual shelter, but also act as a base for psychological security, protection and assistance as individuals age (Snow, 2013). In this sense, the particular interest in attachment to proximal variables (e.g. family members, home and the relevant symbols) could have become an important predictor for making a positive environment clearer into real life.

### Geographical living environment

The geographical living environment in this context mainly refers to the satisfaction of the older adults with the environment in the narrowest sense. During the interview, it was found that those that moved to nursing homes nearby to where their family members resided expressed an element of happiness and life satisfaction.

In particular, the older Chinese have a strong desire to live nearby to their adult children and grandchildren enabling them to stay in contact, and also as a way of reducing the risk of being alienated by those who they are emotionally close to, and value highly. The feelings evoked by living closer to loved ones was likely to produce more positive emotions:

“This care home is very close to my family home. It’s very convenient for me if I want to go back home to get something I need. Also, my adult children could visit me easily, and bring me delicious homemade food as long as they have time. I like living here.” Female, 85 years. Chinese.

Similarly, this was commonly found in older Danes, who preferred to choose care homes close to where their family members resided for the very reason that it would be easier for them to continue receiving attention from those who cared for them previously, and thereby experience more happiness:

“No. I came here this year. It was because my daughter, son-in-law and my granddaughter and her husband and family live here. My daughter thought that now was the time to move to this city, as last winter I had a bad cold and the like when I was alone up “there”. … So it’s also because I'm happy to be here.” Female, 94 years, Dane.

### Institutional environment

It can be seen from the interviews that a warm institutional environment, which has brought a lot of benefits and convenience, is conducive to improving the quality of life of the elderly. The satisfaction and requirements of this group are also directed to the optimization of the policy environment to a large extent. Older adults had feelings of being cared for by the services provided by care homes, as recounted by one female resident:

“I am happy living here. We don’t have to visit doctors in hospitals for them to prescribe if we need regular medicines for our illnesses. Because of the new policy, now we can directly buy medicine from here. It’s pretty convenient for us.” Female, 91 years. Chinese.

Likewise, despite being unsatisfied with the services provided by municipality, especially when taken into account with how they are treated, the older resident still held a positive attitude regarding the care home after his relocation. “*But one thing I know is that after I have come here (to the care home), life seems to be worth much than before*.” He further expanded: *“Yes, I think it is great. And should I recommend [a care home] to someone, then it it's definitely [name of the care home]. Apart from the way I was treated by the municipal employees, I have nothing negative to say.”*

Some appreciated a safe environment that offers protection from falls and injuries. Others evoked the importance of the elements of facility design for their living conditions. These elements could be facilitators in fostering older residents’ well-being in care homes.

### Positive capability

The third main theme that was identified by older residents as contributing to quality of life was from the perspective of one’s capabilities, which can be analyzed into three distinct subthemes: physical health, enjoyment of food and learning to compromise. There is long tradition for maintaining that the primary aim of human activities is happiness, in a sense that goes beyond pleasure and material welfare – that it consists not merely in enjoyment, but that people can “function well in life” (for a recent defence, see Kraut, 2007). And various functions are perceived as highly associated with individuals’ wellbeing. Hence, it seems warranted to assume that functions may play a role in highlighting the importance of positive capability, ahead of obtaining information from the participants (this, and the reflective awareness that it is an assumption on part of researcher, just as much as an empirical fact, can be seen as an example of the partially hermeneutic character of the study, though functions were mentioned by the participants without having been explicitly asked about).

### Physical health

Most of the older adults interviewed highlighted that as they grow older, they are well aware of the obvious changes that occur in their physical strength, memory, reflexes, resistance and the like, and therefore the need to pay more attention to their own maintenance. The premise of a good life seems to be to have a healthy body and the need to work hard to achieve it. Some of the Danish older adults candidly mentioned the importance of physical health when answering questions about their perceptions on the quality of life.

“I feel like that. I haven't been severely sick or anything like that, in that sense, other than ordinary colds and such minor issues. That is, I have never had things that have been very serious.” Female, 94 years, Dane.

“I have never minded getting old if you are healthy. … Yes, it’s probably there, if you think deeper about it. But I don’t think so just I can think of it. Health, that's probably the most important thing.” Male, Dane.

While they concede that their bodily health steadily declines, they are more focused on the fact that they still retain their mentally capabilities. This accords with research by Schnittker (2005), which found that older adults’ self-rated health corresponded more strongly with mental health symptoms such as depression, and less strongly with physical health conditions such as functional limitations and various chronic conditions. Likewise, physical health can be important to the Chinese older adults too; however for them, it is given less priority than other factors. In their replies, it was a happy family life that played a pivotal role in their perception of a quality life together with whether their children were filial to them. This included their adult children and grandchildren and encompassed involvement in their wellbeing (their health and how well they were doing with their work, life and so forth).

“[A] good life? To me, a good life is I hope that the children and grandchildren in the family will be in good health. Nothing bad happens, then I would feel very happy. Well, now that I am older, I hope that the children will be in good health. I hope they are peaceful and filial to me, so that I would be very happy.” Female, 91 years, Chinese.

On the downside, one female resident spontaneously burst into tears when asked what a good life meant to her, and elaborated with the fact that she had lost connection with her daughter and how that deeply disturbed her.

### Enjoyment of Food

The interviews conducted in the Chinese group showed that the ability to continue enjoying food mattered to the Chinese older adults in terms of contributing to the quality of life in care homes. Both how much they can eat, and the quality of food, were highlighted as being important. As one 90-year-old female Chinese resident said “*To me, a good life also means you still can eat, because it means a very good fortune. Many older people, like my age, cannot eat, because of the diseases they suffer in one form or another*.” The ability to eat is seen as an indication of the general “trajectory” of life, of whether things go more or less well overall. Additionally, some viewed meals as one of the most important daily activities in care homes. “*I am single, so I don’t have to be anxious about anything else. My daily activities here are simple, focusing on eating, sleeping and watching TV*” as recounted by a male Chinese care resident. Another elderly female even depicted mealtimes as part of her daily expectations (about having something to look forward to) and thus contributing to her daily happiness with “*the moment going to canteen to enjoy delicious meals there*,” and “*we have a quite good chef here. I am very satisfied with the food he makes every day*.”

Similarly, some Danish participants seemed to share their Chinese peers’ views in terms of enthusiasm for mealtimes. While there may be limitations in their activities compounded by changes in physical mobility, for some, they remained satisfied with their lives if they could compensate it with something else such as good food.

“But, I still have something to do here. I live here where we get something good and indeed a lot, to say at least, for my age. Also, I have always liked some good food and such, and made myself good food and things like that.” Female, 94 years, Dane.

Another male Danish resident was even more direct and replied with “…*yes there needs to be good food*.” “*.[…] and look how hard they [the caretakers] work. […] There’s nothing here [to complain about]…and we get such good food. Although I am no longer as good as eating as I used to be.[…] My mouth becomes dry.”* in responding to what quality of life means to them. Although there may be different perceptions as to what constitutes good food, it nonetheless seems to play a key role in life quality for the majority of people.

### Learning to compromise

The quality of life in nursing homes, when centered on harmony and consensus, seems to require the ability of compromise, be it through shared cultural habits or a more active social construction.^5^ This category was mentioned extensively among residents in the care homes. Due to the emphasis on collectivism in Chinese culture, Chinese elderly residents tended to be more cooperative, compromising and non-confrontational to either protect their social identities (not losing face) or to maintain harmony within the care home (not bothering others). They often tolerated others, and adapted their own behaviours and attitudes to the new established norms and routines of the existing group, in order to avoid the emotional distress caused by potential conflicts with other residents. They also held the belief that harmony in the home is likely to increase their positive emotions on a daily basis. An older female described this scenario in the following excerpt:

“My roommate is aged 90 plus. People normally at this age are nice and kind, but she is aggressive and often speaks bad words to me. Sometimes she makes noises while I am sleeping. I feel unhappy. But whenever I think about it, she is older than me, so I try to convince myself to tolerate for harmony sake. Also, life is not easy for such an old age. We should give her more empathy, less complaints.” Female, 86 years, Chinese.

The Chinese older adults tend to regulate their emotions and cognitive state by suppressing their inner voice or hiding their emotions (not sharing negative feelings such as worries with others) and by dismissing any discomforts with the attitude “*we are useless to the society anyway. Forget it, cherish everyday as a blessing to us at this age*.” Otherwise, they dismissed any problems altogether by not even mentioning them. This equally applies to the Danish participants, who preferred to shy away from conflicts by making no complaints, in order to achieve their own goal: following the social rules while living in a collective life. The phrase “*I cannot complain*” is repeatedly mentioned among participants. A Danish male resident recounted: “… *whether I’m sitting on a chair or on this… As long as I can go to bed myself and get up again, I cannot complain.” “I do not complain about being visited, but it is also entertainment when they do visit*.”

By quietly accepting their current status quo, they rationalize their behaviours to fit in, and in some cases, tolerate behaviours that would previous be seen as unacceptable such as disturbances from their co-residents. In the Danish group, an old lady recalled an occasion when another resident entered her room without knocking on the door which had made her feel rather uncomfortable. She had to accept it as the status quo, despite her reservations. The reasons for this could be lack of trust in others or the invasion of privacy.

Another example is an older male who lived with nine women in a section of a care home. He described how during discussions, the women tended to start quarrelling; as a coping strategy, he had quickly learned to avoid getting involved in these often petty discussions.

Interviewer: “So you’re getting along well with the other residents?”

Older resident: “Yes, yes. We are ten residents. I am the only male [laughs] … Yes, so I have to up to be careful with my responses sometimes. … Otherwise … it works well. At least I think so myself. That squabbling, I don’t give much for that.”

Interviewer: “So the others squabble (kævler)?”

Older resident: “Yes, they damn squabble. I have no interest in that.”

Interviewer: “So you just keep out of that?”

Older resident: “Yes, I just keep out of that.”

This example could also reflect a notion among the older adults that they *ought* to have nothing to complain about, whether it be health-wise or regarding their surroundings. Such perceptions also indicate that a sense of “learned helplessness” can occur when older adults are exposed to inescapable situations in which they gradually lose control of their lives, or fail to achieve desired outcomes. But as highlighted previously, it could also be viewed as a positively adaptive mechanism that help them achieving hedonic goals even as they grow older.

### Positive experience

Positive experience was the fourth dominating theme to emerge when older residents were asked about their quality of life in nursing homes. It encompasses both the actual positive emotion experienced and the level of the interest generated from things that provide novelty, pleasure, comfort, happiness, invigoration, or warmth, including previous life experience, agency, respect and emotional health. While it must be taken into account that the particular interest in the role of emotions in wellbeing that comes from the theoretical background of the study could have played a role in making positive experience stand out so clearly, the tendency to emphasize it was significant and not due to any explicit priming or particular mentioning from the interviewer.

### Previous life experience matters

Older adults in this subtheme used past perspectives to contextualize what their expectations of what a quality of life should be. Some Chinese participants referred to past experiences of their harmonious relationship with family members as a spiritual foundation which positively supported their current life quality in care homes. They frequently shared or reminisced about those precious memories in conversations with family members, which significantly aroused positive feelings and a sense of security in the older people. Even in the present time, situations where family members regularly visited or occasions when they are taken back to their homes on traditional holidays can greatly contribute to a better quality of life. In addition, those who had experienced previous hardships in life were more likely to have a relatively positive appreciation of happiness and appraisal of their life in the present, inasmuch as the hardships they had overcome had provided a historical backbone for comparison. An elderly male resident said: “*I cherish today’s life very much, which is a hundred times better than what I experienced at a younger age, due to too many difficulties at the time*.”

Similarly, in the Danish group, reminiscing about old times seemed to profoundly boost the older adults’ contemporary moods. When a female resident was asked whether or not she still thinks about those previous life experiences and whether they made her happy, she responded with: “*Yes, I do often. Often when I sit and think, something comes out and then I wonder if they still exist and, you know, such different things*.” Some residents also mentioned that the frequent visits by family members profoundly boosted their moods, not only as it reminded them about common past experiences, but because it sometimes allowed them to “relive” them. This can be partially explained by the continuity theory, which suggests that when making adaptive choices, older adults attempt to maintain a certain continuity in their behaviors, values and attitudes, and to preserve a sense of self by adopting strategies which tie the past to the present (Atchley, 1989, 2000). In addition, George (1996) emphasized that the frequency of exposure to previous life events had a lasting impact on one’s subjective well-being and was reflected in attitudes, behaviors and the process of meaning-making in later years.

### Agency

The provision of various activities that interest and entertain the elderly, and the support provided to attend these events, were perceived as crucial factors to improving the quality of life in nursing homes. Some Chinese residents revealed that they had developed new interests, such as playing majong, after moving into a care home, in order to maintain their mental quick-thinking. Others helped organize activities to help others in the nursing homes. Many regarded this as having a positive impact on their own well-being, as helping others brought them pleasure and a sense of purpose to their lives. A female resident recalled an activity she participated in:

“Before the pandemic, our nursing home regularly organized for young people such as university students to come to talk to us. We were very happy to share our previous experiences with them. We had a sense of being recognized and worthy to the society. We are still useful.” Female, 85 years. Chinese.

“If we care and help others when they are in need, they feel the warmth and are happy. We are happy, too.” Female, 84 years. Chinese.

In the Danish group, the interviews showed that most of the elderly people expressed that they would continue to participate in more recreational activities in the elderly care institutions, both to contribute to their positive emotional-being, and as a means of continuing previous life experiences. A 75-year-old Danish female recalled how helping others contributed to her enthusiasm towards life in a care home: *“… And it’s helping others that helps me so much. … I have arranged trips for many others, and that was very fulfilling for me. Also they came to me to have it planned because it meant that they trust me*.” This was perceived by both the Chinese and Danish groups as a significant source of adding meaning to their lives when in care homes. It seems that life quality is associated with engagement with meaningful activities. They were highly motivated by these activities which enabled them to continue their current lifestyles in a positive fashion and gave them an awareness of the value of existence. A similar observation was made by Foster & Walker (2015), who emphasized having one’s own activities as a significant element in active aging and maintenance of health.

### Respect and Emotional health

The older residents pointed to life quality was more valued if respect was shown by the staff, demonstrated by taking meticulous care of their needs, and less so about them acting according to principles. That is, they appreciated not only having their physical care needs met, but also any emotional needs. Additionally, it seems that emotional well-being was an important indicator in reflecting the quality of life experienced by the elderly. Most elderly residents say that living in a care institution is more enjoyable, although there are some who have a more negative attitude. The following quotes demonstrate this, and were expressed in a pretty despondent tone:

“No. They say they're following up, but nothing's going to happen.” Male, 68 years, Dane.

“No… they take no matter who it is. And they distribute the work out there. There is nothing special.” Male, 95 years, Dane.

For the elderly, this does not necessarily mean that considerable effort in assisting them or taking care of them is required, because what they desire the most is more emotional attention from others, rather than purely material support. This is partly explained by the socioemotional selectivity theory which says that older adults tend to focus on a few key relationships as it helps them regulate their emotions better and maximizes any positive effects (Carstensen, 1992).

“They are like my children. There are several times when I feel uncomfortable. They would come and show concern to me, asking whether I need any help.” Female, 85 years. Chinese.

In some cases, meeting and understanding their specific requests can also boost the residents’ mood. An example of this is having special meals prepared in accordance with the residents’ preferences, as recounted by one resident:

“I like certain dishes. Sometimes I ask them if they can cook the dishes for me, they would do it, which makes me very happy.” Female, 91 years. Chinese.

While living in an elderly care institution, with the companionship of peers, can reduce the loneliness of the elderly, some older residents tend to purposefully minimize their interactions with others. Together with the lack of emotional contact from their family members, this can serve to exacerbate feelings of loneliness. It has been challenging for these residents to integrate into collective life, which could be viewed as a consequence of their own personality or choices which may have been influenced by previous experiences. “*For many older people, it seems that very suddenly they are no longer in focus. … The question is what kind of a person you are. Whether you seek out other people and want to be something to them*.” as recalled by one elderly Danish female resident.

This again echoes the theme of “*positive attitude as a good start*” in which those who show a willingness to move tended to hold a more positive attitude, which was particularly instrumental to the process of a fulfilled and satisfied adaptation, as opposed to the less willing group, who held onto a desire to be attached to their previous home or lifestyle – be it physically or emotionally.

## Limitations

There are a number of limitations to the present qualitative study. Firstly, the results were based on a relatively small sample size. It could also be criticized for the lack of geographic diversity, creating a risk that the participants’ views may not represent that of older adults living in other facilities. Thirdly, as all the participants were selected by managers in the care home and had to consent themselves, some selection bias has been unavoidable. Lastly, the findings of the present study might also not be transferable and so the external validity might be questionable. On the other hand, qualitative studies may have their own standards of generalizability and replicability (Nowell & Albrecht, 2018). Detailed descriptions and interpretations were provided to give other researchers a full insight into how the data has been analyzed, but they are undoubtedly influenced by particular interests. The primary aim has been to explore the understandings of older adults from a cultural and subjective perspective to gain in-depth knowledge about how they perceive their life quality and the way it is related to the transition to a care home. Therefore, it can be assumed that the limitations do not render the findings and analyses insignificant, although further research is called for, not least a comparative study that explores the conceptions of life quality in more heterogeneous groups.

## Conclusion

To our knowledge, previous comparative studies exploring the quality of life of older adults in care homes from their own perspectives in China and Denmark are relatively sparse. A key finding from this research was that older care residents in both countries shared a high level of similarity in their perceptions of quality of life, despite living in different cultural settings.

Socio-cultural values may nevertheless have a persistent impact on older adults’ perceptions of quality life in care homes. In this study, older adults considered quality of life as a dynamic process in pursuit of a harmonious state, centering on “change” as the core value, which might be different from stability, confirming or at least compatible with a process-oriented approach to older adults wellbeing (Klausen, 2018), and also confirming the assumption that older adults may have, and live by, particular understandings and perceptions of quality of life (Klausen, 2019). The foundation of Chinese culture is maintaining harmony – where family harmony as a core social unit is placed as a high priority (Chen, 2016), with filial piety playing a pivotal role in family harmony, and it mainly centers around mutual responsibility, respect and emotional care between older parents and their adult children. In terms of collectivism, Chinese older adults have been stereotypically viewed as a homogeneous group who valued the importance of collectivity over individuality, without legitimate autonomous interests (Kuo & Kavanagh, 1994).

Nevertheless, findings of this study showed that these same values of harmony were present in the perceptions and understanding of Danish older adults as well, although they were sometimes expressed in a different form, such as at the level of social systems. Hence the assumption that cultural differences influence perception of quality of life was partly met, but also partly challenged; some cultural differences turned out to be more superficial. On the surface, creating harmony may be viewed as a potent solution to stimulate positive emotions, as it might protect their self-esteem. However, it may not be a remedy for solving core problems, but rather a means of shunning them. This may partly explain why older adults prefer to relocate to a care home near their family members, as family may impart a balanced sense of hope even though they are "harmonized" by society systems. Compared to other social relationships, the family bond may entail a better soil for cultivating love and the other natural affection that should be at the center of our lives, and at the same time, provide them with a sense of extended existence and fulfilment. Additionally, the ideal of family closeness may be more desirable for older adults in maintaining mental health.

In the context of harmony, adaptive processes were adopted as coping tools by older adults in order to enhance their life quality. Quality of life in the transition to care homes was seen as characterized by having a positive attitude as a necessary basis combined with the ability to compromise. Although attitude change is difficult to achieve, it seems to be very important for securing wellbeing in the transitory phase and after, and is known that it can be fostered through strategies of communication, such as political speeches and advertisements, as demonstrated in the reinforcement theories of attitude change (Deaux et al., 1993).

In terms of harmonious pursuit, older adults’ perspective on life quality could be seen as integrating both simple and complex not-conflicting values. Considerable weight was given to apparently small daily pleasures such as enjoyment of food, elation due to family member visits, and contentment with tailored care provided by staff. All these small pleasures appear to be strongly enhanced in complex environments, such as a warm family, institution and society, which are all potentially interlinked. Irrespective of the particular path they followed, be it finding comfort in memories, developing good relationships with others, participating in activities or acquiring respect from others, such activities provided the older adults with a more balanced sense of completeness and peace of mind.

Findings from this study also corrects the common misconception that often depicts older adults in individualistic culture as active and diverse care recipients, with the Chinese as a more homogenous group consisting of passive, vulnerable, conformist and/or dependent subjects. In the latter stages of life, the Chinese appeared more like equal human beings who expressed and interpreted their own understanding of what the quality of life should be like, within particular social structures.

The present study has implications for developing policies in the future, including tailored interventions, and meeting the unique needs of older adults, and thus improving their wellbeing. The findings suggest that family relations in the form of emotional bonds remain important and can also be maintained under and after the transition to a care home, and under conditions of modern life (Author and colleagues in press). The findings also indicate that the quality of the very transition phase matters significantly; positive and negative experiences related to past life stages or events continue to influence perceived quality of older adults, to a larger extent than recognized by standard approaches to wellbeing and aging (and thus again confirm the holistic and process-oriented approach; though prior sympathy for this approach may of course to some extent have influenced the interpretation). The study also indicates that further research is required that considers the values of harmony as the central role of older adults’ perception of life quality. It also seems important to conduct future comparative studies with the use of diary studies that were followed up by a second interview to enrich data analysis and substantiate the findings from the present study.

## Footnotes

1 Lin (2011) revealed that tensions between intergenerations with respect to family support might become catalysts for issues for older adults considering alternatives such as institutional care in urban China.

2 Yap (1969) was the first to describe these untranslatable mental disorders as “culture-bound syndrome,” that seemed to occur only in certain societies, in contrast to the usual mental illness that was experienced universally. Then Bhugra & Jacob (1997) suggested that these culturally bound behaviors were usually diagnosed with a limited appreciation of certain socio-environmental influences.

3 Chuang & Abbey (2009) portrayed older adults relocating to care homes as those who feel “cut off the root”.

4 This mainly refers to the harmony of the “social welfare system,” as described by Jespersen (2004) where a prerequisite for its existence is collectively striving to create values such as “uniformity and conformity, innate solidarity, and exclusivity with regard to the outside world”.

5 Cultural habits mainly refer to how Chinese older adults maintain harmony among themselves, family members and society. However, more emphasis may be given to the family and how to avoid not being a burden to them. From the perspective of social consensus, the older Danes may point towards to not being a burden to society. This echoes the findings of Andersson et al (2008) who stated that the fear of being a burden encouraged older adults to hide their real feelings or suppress their inner voice, to maintain a façade of normality.

## References

[CR1] Albertini M, Kohli M, Vogel C (2007). Intergenerational Transfers of Time and Money in European Families: Common Patterns – Different Regimes?. Journal of European Social Policy.

[CR2] Andersson M, Hallberg IR, Edberg A (2008). Old people receiving municipal care, their experiences of what constitutes a good life in the last phase of life: A qualitative study. International Journal of Nursing Studies.

[CR3] Atchley RC (1989). A continuity theory of normal aging. The Gerontologist.

[CR4] Atchley, R. C. (2000). Continuity and Adaptation in Aging: Creating Positive Experiences. Baltimore, Maryland: Johns Hopkins University Press.

[CR5] Baltes PB, Smith J (2003). New Frontiers in the Future of Aging: From Successful Aging of the Young Old to the Dilemmas of the Fourth Age. Gerontology.

[CR6] Bekhet AK, Zauszniewski JA (2014). Individual characteristics and relocation factors affecting adjustment among relocated American and Egyptian older adults. Issues in Mental Health Nursing.

[CR7] Bengtson, V. L., Lowenstein, A., Putney, N., & Gans, D. (2003). Global aging and the challenges to families. In V.L.Bengtson & A. Lowenstein (Eds.), *Global aging and challenges to families* (pp.1–24). Hawthorne, NY: Aldine de Gruyter

[CR8] Bergland A, Kirkevold M (2005). Resident–caregiver relationships and thriving among nursing home residents. Research in Nursing & Health.

[CR9] Bhugra D, Jacob KS, Bhugra D, Monro A (1997). Culture bound syndromes. Troublesome Disguises.

[CR10] Brandtstädter, J. (2015).” Adaptive resources of the aging self: Assimilative and accommodative modes of coping”. In N. A. Pachana (Ed.), *Encyclopedia of Geropsychology*. Singapore: Springer Singapore, 1–8

[CR11] Carstensen LL (1992). Motivation for social contact across the life span: A theory of socioemotional selectivity. Nebraska Symposium on Motivation.

[CR12] Chang Y-P, Schneider JK (2010). Decision-making process of nursing home placement among Chinese family caregivers. Perspectives in Psychiatric Care.

[CR13] Chen L (2011). Elderly Residents' Perspectives on Filial Piety and Institutionalization in Shanghai. Journal of Intergenerational Relationships.

[CR14] Chen GM, Jensen KB, Craig RT (2016). Harmony theory (Chinese). International encyclopedia of communication theory and philosophy.

[CR15] Chen, S. Y. (1996). Social policy of the economic state and community care in Chinese culture: Aging, family, urban change, and the socialist welfare pluralism. Avebury: Brookfield.

[CR16] Chu L-W, Chi I (2008). Nursing homes in China. Journal of the American Medical Directors Association.

[CR17] Chu CY, Xie Y, Yu RR (2011). Coresidence With Elderly Parents: A Comparative Study of Southeast China and Taiwan. Journal of Marriage and the Family.

[CR18] Chuang YH, Abbey J (2009). The culture of a Taiwanese nursing home. Journal of Clinical Nursing.

[CR19] Cooney A, Murphy K, O’Shea E (2009). Resident perspectives of the determinants of quality of life in residential care in Ireland. Journal of Advanced Nursing.

[CR20] Custers AFJ, Westerhof GJ, Kuin Y, Gerritsen DL, Riksen-Walraven, & J.M.  (2012). Relatedness, autonomy, and competence in the caring relationship: The perspective of nursing home residents. Journal Aging Studies.

[CR21] De Bellis A (2010). Australian residential aged care and the quality of nursing care provision. Contemporary Nurse.

[CR22] Deaux, K., Dane, F. C, & Wrightsman, L. S. (1993). Social psychology in the 90s. Pacific Grove, CA: Brooks/Cole.

[CR23] Deci EL, Ryan RM (2008). Hedonia, eudaimonia, and wellbeing: An introduction. Journal of Happiness Studies.

[CR24] Delle Fave A, Brdar I, Freire T (2011). The Eudaimonic and Hedonic Components of Happiness: Qualitative and Quantitative Findings. Social Indicator Research.

[CR25] Diener E, Oishi S, Lucas RE, Snyder CR, Lopez SJ (2002). Subjective well-being: The science of happiness and life satisfaction. Handbook of Positive Psychology.

[CR26] Emiliussen J, Engelsen S, Christiansen R, Klausen SH (2021). We are all in it!: Phenomenological Qualitative Research and Embeddedness. International Journal of Qualitative Methods.

[CR27] Eurostat. Population structure and ageing. (2016). Retrieved from http://ec.europa.eu/eurostat/statistics-explained/index.php/Populationstructureandageing. Accessed Jan 2021

[CR28] Feng Z, Zhan H, Feng X, Liu C, Sun M, Mor V (2011). An Industry in the Making: The Emergence of Institutional Elder Care in Urban China. Journal of the American Geriatrics Society..

[CR29] Foster, L., & Walker, A., (2015). Active and successful aging: A European policy perspective. *The Gerontologist, 55*(1), 83–90. 10.1093/geront/gnu028 10.1093/geront/gnu028 PMC4986585 24846882

[CR30] George L (1996). Missing links: The case for a social psychology of the life course. The Gerontologist.

[CR31] Gu L, Rosenberg MW, Zeng J (2017). May/June). Changing caregiving relationships for older home-based Chinese people in a transitional stage: Trends, factors and policy implications. Archives of Gerontology and Geriatrics.

[CR32] Haybron, D. (2008). *The Pursuit of Unhappiness*. Oxford, England.: Oxford University Press.

[CR33] Hernandez, G. B. R., Murray, C. M., & Standley, M. (2020). An intergenerational playgroup in an Australian residential aged-care setting: A qualitative case study. *Health and Social Care in the Community*, 1-10. 10.1111/hsc.13149 32852104

[CR34] Higgs, P., & Gilleard, C. (2015). Rethinking old age: Theorising the fourth age. London: Palgrave Macmillan.

[CR35] Jespersen, KJV (2004). *A History of Denmark*. Palgrave Macmillan.

[CR36] Johnson, R. A., & Bibbo, J. (2014, August). Relocation decisions and constructing the meaning of home: A phenomenological study of the transition into a nursing home. *Journal of Aging Studies,* 30(August), 56–63. 10.1016/j.jaging.2014.03.005 10.1016/j.jaging.2014.03.005 PMC4099410 24984908

[CR37] Jongenelis K, Pot AM, Eisses AMH, Beekman ATF, Kluiter H, Ribbe MW (2004). Prevalence and risk indicators of depression in elderly nursing home patients: The AGED study. Journal of Affective Disorders.

[CR38] Kahneman D, Krueger AB, Schkade DA, Schwarz N, Stone AA (2004). A survey method for characterizing daily life experience: The Day reconstruction method. Science.

[CR39] Kaneko Y, Motohashi Y, Sasaki H, Yamaji M (2007). Prevalence of depressive symptoms and related risk factors for depressive symptoms among elderly persons living in a rural Japanese community: A cross-sectional study. Community Mental Health Journal.

[CR40] Kao HF, Travis SS, Acton GJ (2004). Relocation to a long-term care facility: Working with patients and families before, during, and after. Journal of Psychosocial Nursing and Mental Health Services.

[CR41] Kehyayan V, Hirdes JP, Tyas SL, Stolee P (2015). Residents’ self-reported quality of life in long-term care facilities in Canada. Canadian Journal on Aging/la Revue Canadienne Du Vieillissement.

[CR42] Keyes C. L. (2005). Mental illness and/or mental health? Investigating axioms of the complete state model of health. *Journal of consulting and clinical psychology, 73*(3), 539–548. 10.1037/0022-006X.73.3.539 10.1037/0022-006X.73.3.539 15982151

[CR43] Keyes CLM (2007). Promoting and protecting mental health as flourishing: A complementary strategy for improving national mental health. American Psychologist.

[CR44] Klausen, S. H. (2018). Ethics, knowledge, and a procedural approach to wellbeing. *Inquiry*, 1-17. 10.1080/0020174X.2018.1529619

[CR45] Klausen, S. H. (2019). Understanding Older Adults' Wellbeing from a Philosophical Perspective. *Journal of Happiness Studies*.

[CR46] Kraut, R. (2007). What is Good and Why. The Ethics of Well-Being. Cambridge, MA: Harvard University Press

[CR47] Krizaj T, Warren A, Slade A (2018). “Holding on to what i do”: Experiences of older Slovenians moving into a care home. The Gerontologist.

[CR48] Kuo CL, Kavanagh KH (1994). Chinese perspectives on culture and mental health. Issues in Mental Health Nursing.

[CR49] Lee DT (1999). Transition to residential care: Experiences of elderly Chinese people in Hong Kong. Journal of Advanced Nursing.

[CR50] Lee DT, Woo J, Mackenzie AE (2002). The cultural context of adjusting to nursing home life: Chinese elders' perspectives. The Gerontologist.

[CR51] Meeks S, van Haitsma K, Kostiwat I, Murrell SA (2010). Positivity and well-being among community-residing elders and nursing home residents: What is the optimal affect balance?. Journal of Gerontology.

[CR52] Morgen M (2010). Ældrestyrken. Mandag Morgen.

[CR53] National Statistical Bureau of the People’s Republic of China. (2019). Statistical communique on national economic and social development. http://www.stats.gov.cn/tjsj/zxfb/202001/t20200119_1723767.html. Accessed Jan 2021

[CR54] Nowell B, Albrecht K (2018). A Reviewer’s Guide to Qualitative Rigor. Journal of Public Administration Research and Theory.

[CR55] Ohrnberger, J., Fichera, E., & Sutton, M. (2017). The relationship between physical and mental health: A mediation analysis. *Social science & medicine (1982), 195*, 42–49. 10.1016/j.socscimed.2017.11.008 10.1016/j.socscimed.2017.11.008 29132081

[CR56] Palmore EB (1983). Cross-cultural research: State of the art. Research on Aging.

[CR57] Park N, Peterson C (2009). Achieving and sustaining a good life. Perspectives on Psychological Science.

[CR58] Patton, M. Q. (2002). *Qualitative research and evaluation methods* (3^rd^ ed.). Sage.

[CR59] Pavot W, Diener E (2008). The Satisfaction with Life Scale and the emerging construct of life satisfaction. Journal of Positive Psychology.

[CR60] Ron P (2004). Depression, Hopelessness, and Suicidal Ideation Among the Elderly. Journal of Gerontological Social Work.

[CR61] Ryan RM, Deci EL (2001). On happiness and human potentials: A review of research on hedonic and eudaimonic wellbeing. Annual Review of Psychology.

[CR62] Schnittker J (2005). When mental health becomes health: Age and the shifting meanings of self-evaluation of general health. Millbank Quarterly.

[CR63] Seligman, M.E.P. (2011). Flourish. New York: Free Press.

[CR64] Sen, A. (1999). *Development as Freedom*. Oxford, England.: Oxford University Press.

[CR65] Smith, J. A. (2009). Interpretative phenomenological analysis: Theory, method and research. London, U.K: SAGE Publications Ltd.

[CR66] Smith, J. A. (2015). Qualitative psychology: A practical guide to research methods. London, U.K: SAGE Publications Ltd.

[CR67] Snow, E. L. (2013). The impact of attachment on friendship satisfaction and correlates of well-being of older adult females. California State University, Long Beach.

[CR68] Stathi, A., & Simey, P. (2007). Quality of life in the Fourth Age: Exercise experiences of nursing home residents. *Journal of Aging & Physical Activity*, 15(3), 272–286. Retrieved from http://opus.bath.ac.uk/6271/1/Stathi_JAPA_2007_15_272286.pdf. Accessed Jan 2021 10.1123/japa.15.3.272 17724394

[CR69] Sullivan GJ (2017). Older adults transiton to long term care: A meta synthesis. Journal of Gerontological Nursing.

[CR70] Tapper, J. (2019). How the Elderly Can Help the Young – and Help Themselves. *The Guardian* 05.01. 2019. https://www.theguardian.com/society/2019/jan/05/children-eldery-intergenerational-care-advantages. Accessed Mar 2021

[CR71] Taylor, Charles. (1992). The Ethics of Authenticity. Cambridge: Harvard University Press.

[CR72] Tuckett AG (2007). The meaning of nursing-home: ‘Waiting to go up to St. Peter, OK! Waiting house, sad but true’—An Australian perspective. Journal of Aging Studies.

[CR73] Urry HL, Nitschke JB, Dolski I, Jackson DC, Dalton KM, Mueller CJ (2004). Making a life worth living. Psychological Science.

[CR74] Vang, G. (2020). Kong Gauers Gård. Vi bygger børnehave og plejehjem sammen [we build a combined kindergarten and care home]. https://www.vejle.dk/om-kommunen/udvikling-med-vilje/vi-udvikler-velfaerden/vi-bygger-boernehave-og-plejehjem-sammen/. Accessed Apr 2021

[CR75] Wu B, Carter MW, Goins RT, Cheng C (2005). Emerging services for community-based long-term care in urban China: A systematic analysis of Shanghai’s community-based agencies. Journal of Aging & Social Policy.

[CR76] Wu SC, White A, Cash K, Foster S (2009). Nursing home care for older people in Taiwan: A process of forced choice. Journal of Clinical Nursing.

[CR77] Yan Z, Yang X, Wang L, Zhao Y, Yu L (2014). Social change and birth cohort increase in loneliness among Chinese older adults: A cross-temporal meta-analysis, 1995–2011. International Psychogeriatrics.

[CR78] Yang QH (1988). The aging of China’s population: Perspectives and implication. Asia Pacific Population Journal.

[CR79] Yap PM. (1969). The culture bound syndromes. In: Cahil W, Lin TY (eds). Mental Health Research in Asia and The Pacific. *East-West Centre Press*, Honolulu, 33–53. 4.

[CR80] Yeboah C, Bowers B, Rolls C (2013). Culturally and linguistically diverse older adults relocating to residential aged care. Contemporary Nurse.

[CR81] Yu Z, Yoon JY, Grau B (2016). How do levels of nursing home adjustment differ by length of stay?. International Journal of Nursing Practice.

[CR82] Zimmermann, C., Swami, N., Krzyzanowska, M., Hannon, B., Leighl, N., Oza, A., Moore, M., Rydall, A., Rodin, G., Tannock, I., Donner, A., & Lo, C. (2014). Early palliative care for patients with advanced cancer: a cluster-randomised controlled trial.* Lancet (London, England), 383*(9930), 1721–1730. 10.1016/S0140-6736(13)62416-2 10.1016/S0140-6736(13)62416-2 24559581

